# Common neurocircuitry mediating drug and fear relapse in preclinical models

**DOI:** 10.1007/s00213-018-5024-3

**Published:** 2018-09-25

**Authors:** Travis D. Goode, Stephen Maren

**Affiliations:** Department of Psychological and Brain Sciences and Institute for Neuroscience, Texas A&M University, 301 Old Main Dr., College Station, TX 77843-3474 USA

**Keywords:** Addiction, Amygdala, Bed nucleus of the stria terminalis, Extinction, Hippocampus, Prefrontal cortex, PTSD, Reinstatement, Relapse

## Abstract

**Background:**

Comorbidity of anxiety disorders, stressor- and trauma-related disorders, and substance use disorders is extremely common. Moreover, therapies that reduce pathological fear and anxiety on the one hand, and drug-seeking on the other, often prove short-lived and are susceptible to relapse. Considerable advances have been made in the study of the neurobiology of both aversive and appetitive extinction, and this work reveals shared neural circuits that contribute to both the suppression and relapse of conditioned responses associated with trauma or drug use.

**Objectives:**

The goal of this review is to identify common neural circuits and mechanisms underlying relapse across domains of addiction biology and aversive learning in preclinical animal models. We focus primarily on neural circuits engaged during the expression of relapse.

**Key findings:**

After extinction, brain circuits involving the medial prefrontal cortex and hippocampus come to regulate the expression of conditioned responses by the amygdala, bed nucleus of the stria terminalis, and nucleus accumbens. During relapse, hippocampal projections to the prefrontal cortex inhibit the retrieval of extinction memories resulting in a loss of inhibitory control over fear- and drug-associated conditional responding.

**Conclusions:**

The overlapping brain systems for both fear and drug memories may explain the co-occurrence of fear and drug-seeking behaviors.

## Introduction

The primary goal of cognitive behavioral therapies for psychiatric disorders is to produce long-term therapeutic benefits that improve the quality of life in individuals suffering from disease. For some of these disorders, including post-traumatic stress disorder (PTSD) and substance use disorders, behavioral therapies target the maladaptive memories that underlie, at least in part, pathological conditioned fear responses and drug-seeking behaviors, respectively. These therapies are based on considerable work in both humans and other animals indicating that fundamental associative learning processes, including Pavlovian and instrumental conditioning, contribute to the pathogenesis and comorbidity of anxiety and addiction. For example, associations between trauma-related stimuli (e.g., flashes of light, smells of blood, and loud noises) and their outcomes (e.g., physical pain or fear of loss of life) underlie some aspects of fear-related behavior in patients suffering from PTSD (Rothbaum and Davis 2003; Mahan and Ressler 2012; Lissek and Meurs 2015). Likewise, learned associations in cocaine-dependent patients between drug-related stimuli (e.g., mirrors, razor blades, and dollar bills) and their outcomes (e.g., euphoria) are believed to support some aspects of drug-seeking behavior (particularly early in addiction) (Robinson and Berridge 1993; Di Chiara et al. 1999).

Although cognitive behavioral therapies, such as prolonged exposure therapy, are often successful, some individuals experience only short-term gains and suffer from a relapse of symptoms. Patients with anxiety disorders (Craske et al. 2009) and trauma- and stressor-related disorders (Ross et al. 2017), for example, may exhibit a reemergence of behaviors and symptoms of fear and anxiety that have previously been reduced through therapy (Yonkers et al. 2000; Yonkers et al. 2001; Yonkers et al. 2003). This phenomenon, termed “fear relapse,” undermines the goals of therapy, is unpleasant, and is quite common (Boschen et al. 2009; Vervliet et al. 2013b; Vervliet et al. 2013a): across an 8-year study by Yonkers et al. (2003), the authors documented a cumulative probability of 64% of relapse in women patients treated for panic disorder. Likewise, individuals with substance use disorders who reduce intake and abstain from taking drugs can experience a return of drug cravings that drives them to once again seek and take drugs (O’Brien et al. 1977; McAuliffe 1990; Hser et al. 1993). Consequently, “drug relapse” is a major challenge for long-term rehabilitation programs and puts individuals at risk for overdose (Püschel et al. 1993; Chopra and Marasa 2017). Drug relapse is also extremely common: one report found that 40–60% of patients treated for drug dependence return to active substance use within a year following treatment discharge (McLellan et al. 2000). Given the high cost of mental illness (Warshaw et al. 1993; Souêtre et al. 1994; Rehm et al. 2009; Craske et al. 2017) and the comorbidity of drug use and other disorders (Brown et al. 1999; Kessler et al. 2005; Logrip et al. 2012), it is essential that progress be made towards understanding common behavioral and brain mechanisms of relapsed behaviors. Accordingly, the purpose of this review is to explore current studies of the neurobiology of relapse, to compare and contrast these reports, and to integrate findings in the fields of affective and addiction neuroscience (Peters et al. 2009; Abraham et al. 2014). We derive these insights primarily from preclinical animal research but consider work in humans where appropriate. We first identify and define the terms used in the literature to highlight the various forms of fear and drug return. We will then harmonize these terms to aid in our discussion of overarching neural mechanisms of relapse. In later sections, we discuss current data encompassing the neural circuits of fear and drug recovery, with particular emphasis on overlapping circuits of the amygdala, prefrontal cortex, hippocampus, and bed nucleus of the stria terminalis.

### Extinction of fear and drug-seeking

Cognitive behavioral therapies for trauma and addiction, such as prolonged exposure therapy, are thought to involve mechanisms of *extinction* (Lovibond 2004; Peters et al. 2009; Milad and Quirk 2012; Abramowitz 2013; Craske et al. 2014; Milad et al. 2014; Forcadell et al. 2017; Craske et al. 2018; Everitt et al. 2018). As a result, important features of fear and drug relapse in the clinic can be effectively studied in the laboratory using conditioning and extinction procedures in both humans and animal models. Pavlovian conditioning is a fundamental form of learning by which animals associate stimuli (Pavlov 1927). In the laboratory, this commonly involves one or more presentations of a detectable but harmless stimulus (such as a discrete light or tone; termed the conditioned stimulus, CS) with a noxious stimulus (such as an electric shock; termed the unconditioned stimulus, US). The aversive US elicits numerous behaviors and physiological reactions, including tachycardia, hypothalamic–pituitary–adrenal (HPA) axis activity, and activity bursts (Bolles and Fanselow 1980; Fanselow 1994). After one or more pairings of the CS and US, defensive fear responses will be elicited by the CS alone—freezing often serves as the dependent measure of learning in rodent models. In addition to the CS, the context in which conditioning occurs also comes to evoke conditioned fear after fear conditioning (i.e., contextual conditioning) (Curzon et al. 2009; Luyten et al. 2011; Urcelay and Miller 2014). Fear to the CS (whether a context or discrete cue) can be reduced using extinction procedures, in which the CS is repeatedly presented in the absence of the US (Pavlov 1927; Myers and Davis 2002; Hermans et al. 2006; Myers and Davis 2007; Chang et al. 2009). The extinction of conditioned fear appears to generate a new memory (a CS–“no US” memory) that inhibits and competes with the original conditioned memory of the CS–US association for expression (Bouton 2000; Bouton 2002; Maren 2011; Rosas et al. 2013; Bouton 2014); later, we will highlight the situations in which the original CS–US memory dominates to express relapse.

Conditioning and extinction procedures can also be used to study drug-seeking behavior and its relapse. For example, animals will readily learn to self-administer drugs of abuse when given the opportunity to perform an instrumental (operant) response to obtain the drug (Robinson and Berridge 1993; Robinson and Berridge 2000; Weiss et al. 2001; Feltenstein and See 2008). In typical self-administration paradigms, rats will readily learn to press a lever, pull a chain, or make a nose poke to earn an intravenous drug infusion. In these tasks, learning is indexed by the magnitude and frequency of these responses over time. Thus, unlike Pavlovian fear conditioning, the reinforcer or outcome (“O”; e.g., intravenous drug) is dependent on the behavioral response (“R”; e.g., nose poke) of the animal. Nevertheless, instrumental conditioning involves associative learning: the animal comes to associate its response with the outcome of reward (R–O associations) (Bouton and Todd 2014). Additionally, predictive and environmental stimuli can be used to “set the occasion” for the R–O relationship (Holland 1992; Urcelay and Miller 2014; Khoo et al. 2017). In particular, predictive cues (such as lights or tones), or even particular contexts, can be used to signal when or which behavior to initiate for the reward (Lynch et al. 2010; Chesworth and Corbit 2017); in turn, these stimuli (“S”) can be associated with the learned response (S–R associations). Thus, in the presence of such cues, the cues and drug itself are akin to the CS and US of a Pavlovian conditioning experiment, respectively. Similar to contextual fear conditioning, the environment or context can be associated with the drug US. For example, conditioned place preference (CPP) involves the pairing of drug(s) with a particular environment; the readout of associative learning is then measured by observing the extent to which the animal spends time in or shows a preference for a drug-paired context vs. a location in which no drug was administered (Tzschentke 1998; Roux et al. 2003; Tzschentke 2007). The extinction of drug-seeking behaviors involves omitting delivery of the outcome after the instrumental response is performed. During extinction procedures, animals learn that their behaviors no longer produce the outcome and this degrades the R–O and S–R associations that underlie instrumental drug-seeking behavior. Similar to the extinction of fear, the omission of reward generates an extinction memory that inhibits the expression of drug-seeking behaviors (Millan et al. 2011). After extinction of drug-seeking, there are many factors than can cause this behavior to relapse (Trask et al. 2017; Namba et al. 2018; Marchant et al. 2018). Consequently, mechanisms of extinction appear to have important similarities for conditioned fear and drug-seeking, which may suggest overlapping neural mechanisms.

Of course, Pavlovian and instrumental responses can be reduced through procedures other than extinction. For example, drug dependence can be attenuated via voluntary or forced abstinence [(Venniro et al. 2016); although this is known to incubate the drug-seeking responses] as well as through punishment of the instrumental response (in which the drug-seeking behavior is instead paired with a noxious outcome) (Smith and Laiks 2017; Marchant et al. 2018). The brain mechanisms of relapse may differ as a function of the procedure used to reduce responding (Pelloux et al. 2018). Similarly, fear-conditioned responses can be reduced through counterconditioning procedures in which the CS is paired with a reward rather than the aversive US (Holmes et al. 2016; Kang et al. 2018); and this engages additional neural circuitry (Bulganin et al. 2014; Correia et al. 2016). Furthermore, it should be noted that instrumental tasks in which animals can escape or avoid the aversive US are also important models for the study of the expression and relapse of fear and of avoidance symptoms in PTSD patients (Campese et al. 2016; LeDoux et al. 2017; Campese et al. 2017; Moscarello and Maren 2018). However, fear relapse circuitry in aversive learning has primarily been studied in the context of unavoidable shock (therefore, our review will center on these studies).

### Relapse in the laboratory: harmonizing the terminology

With the aforementioned conditioning and extinction mechanisms in mind, we will now discuss models of relapse. The first reported evidence of relapse of an extinguished conditioned response was discovered by Pavlov (Pavlov 1927). Pavlov observed that an extinguished salivary response could “spontaneously recover” if one of his dogs was tested to the extinguished CS after some delay. Since then, relapse of extinguished fear has been studied extensively, and we now know it can be caused by numerous factors in both humans and other animals (Bouton 2002; Bouton 2004; Bouton et al. 2006; Vervliet et al. 2013b; Vervliet et al. 2013a; Goode and Maren 2014; Bouton 2014; Maren and Holmes 2016). Likewise, relapse or “reinstatement” of drug-seeking behavior has been demonstrated in humans and other animals for many drugs of abuse, including heroin, cocaine, nicotine, methamphetamine, and alcohol (as well as combinations of these drugs) (Wikler 1948; Weiss et al. 2001; Weiss et al. 2006; Stoker and Markou 2015; Marchant et al. 2015; Venniro et al. 2016; Mantsch et al. 2016). However, there has been divergence in the terminology used to describe relapse phenomena by scientists interested in mechanisms of fear learning and those interested in drug addiction, for example. This is important, because the underlying triggers of fear and drug relapse may rely on similar psychological processes and on similar brain circuits (Bossert et al. 2013; Farrell et al. 2018). Furthermore, fear and drug relapse can be triggered by similar events and relapse of one often influences the other (Sanchez and Sorg 2001; Kutlu et al. 2016).

**Fig. 1:**
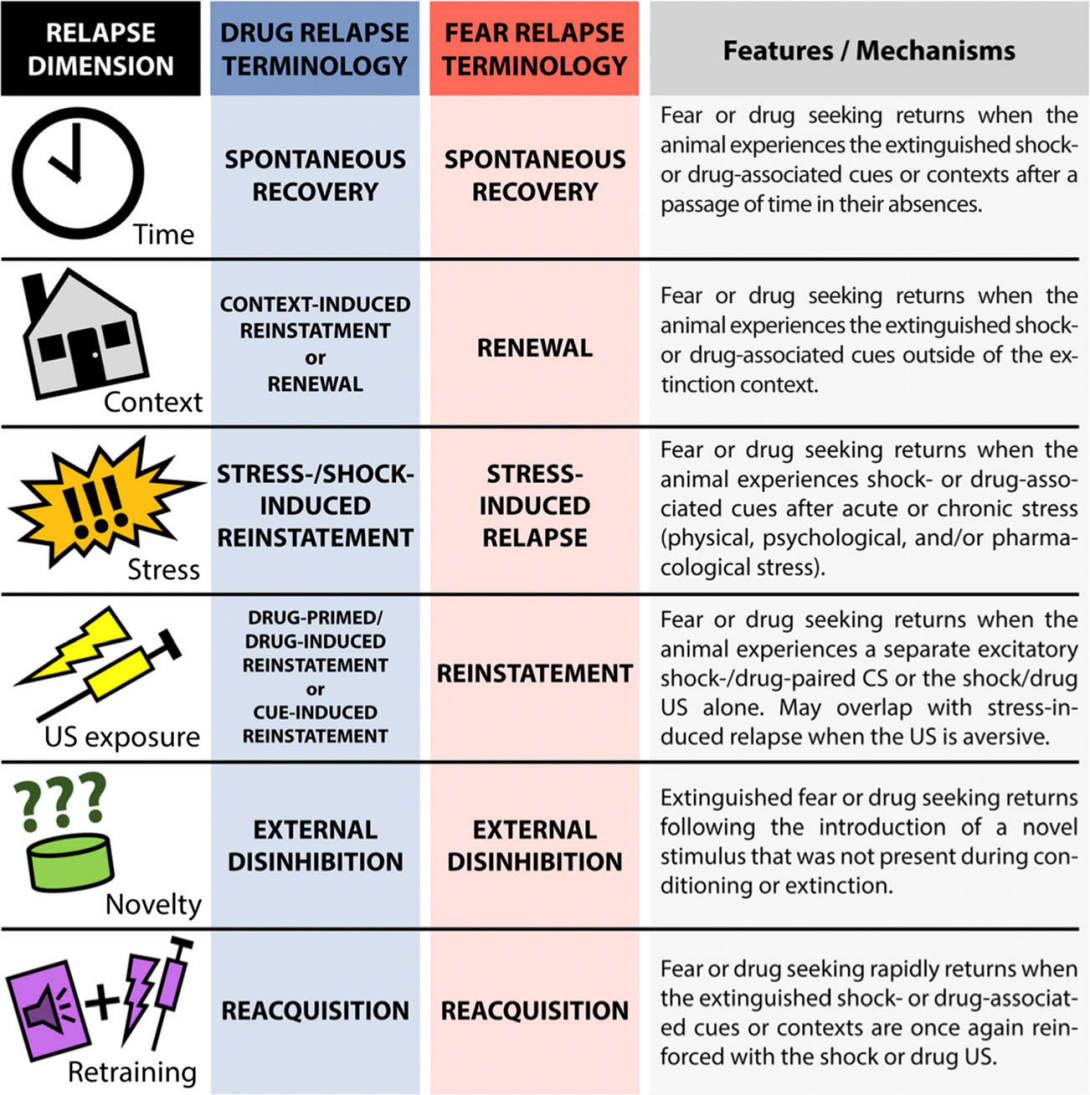
A summary of various drug and fear relapse scenarios, the common and divergent terms used to describe them, and their features

Figure 1 summarizes the major features of various forms of relapse and illustrates the terminology used by learning theorists and addiction biologists to describe relapse. In the fear learning laboratory, *relapse* is defined as a significant and at times long-lasting return of conditional responding to a previously extinguished cue. *Fear relapse* is often used to describe clinical relapse in humans, as well as in laboratory models of humans and other animals. *Return of fear* (ROF) is sometimes used to describe a form of fear relapse in humans that may be experimentally induced but may not necessarily be related to clinical psychopathology, or may simply be less intense (Vervliet et al. 2013a). We will primarily use *fear relapse* in the present review. Several factors can promote the return of extinguished conditional responding. Generally, extinguished fear returns after a context shift (*renewal*) (Bouton and Bolles 1979b; Goode et al. 2017), the experience of the US or other excitatory CSs (*reinstatement*) (Rescorla and Heth 1975; Haroutunian and Riccio 1977; Bouton and Bolles 1979a; Morris et al. 2005a; Halladay et al. 2012; Goode et al. 2015a), and/or a passage of time since extinction (*spontaneous recovery*) (Rescorla 2004). Furthermore, stress exposure (beyond aversive US exposure, and including pharmacological stressors or induction of activity in stress-reactive regions) may also induce relapse (*stress-induced relapse*) (Haroutunian and Riccio 1977; Kellett and Kokkinidis 2004; Morris et al. 2005b; Deschaux et al. 2013; Kinner et al. 2018) or enhance relapse of other forms (Knox et al. 2012). Fear may also return after the extinguished cue is once again paired with the US (*reacquisition*) (Bouton and Swartzentruber 1989). Finally, extinguished fear can return after introduction of a novel stimulus (*external disinhibition*) (Maren 2014; Giustino et al. 2016). Often, relapse of extinguished fear is complete, and returns fear to the level of that expressed by control animals that did not receive extinction training. Note that relapse may also involve new learning/conditioning, especially in cases where the US is re-experienced during reinstatement and reacquisition (Sokol and Lovibond 2012). These phenomena are also observed in appetitive (non-drug-related) conditioning situations; however, we will focus on the return of extinguished fear for the purposes of this review.

In addiction laboratories, a return of instrumental responding is typically referred to as a *reinstatement of drug-seeking*. This is characterized by a return of drug-seeking or taking behaviors following extinction of the original drug-seeking response. The term *drug relapse* is typically reserved for the clinical manifestation of the return of drug-seeking after extinction of drug taking in humans (Hunt et al. 1971). *Drug reinstatement* most often serves as the umbrella term for laboratory models of drug relapse (human or research animal) (O’Brien et al. 1992; Self and Nestler 1998; Leri and Stewart 2002). The mechanisms underlying the return of drug-seeking (e.g., changes in context, stress) are often further clarified by prefacing “drug reinstatement” with the trigger in question (e.g., “context-induced” or “stress-induced” reinstatement). As such, this nomenclature differs from how fear relapse is most often described for laboratory studies (although some researchers will at times make use of such terms as renewal to describe the context-induced return of drug-seeking). As with fear relapse, drug reinstatement may occur in the drug-seeking context (*context-induced reinstatement*) (Crombag and Shaham 2002), the experience of either the drug US, a non-extinguished drug cue, or other stressors (*drug-primed-*, *cue-induced-*, or *stress-induced reinstatement*, respectively) (Shaham and Stewart 1995; Shaham et al. 1997; Crombag and Shaham 2002), and/or the passage of time (*spontaneous recovery*) (Shaham et al. 1997). Post-extinction drug conditioning trials also reinstate drug-seeking (*reacquisition*) (Garcin et al. 1977; Leri and Rizos 2005). Novel cues can also reinstate drug-seeking (*external disinhibition*) (Bastle et al. 2012). Note the differences in the use of “reinstatement” for both fear and drug return.

Given the overlapping mechanisms, but unique terminology, for both fear relapse and drug reinstatement, we will for the sake of consistency utilize a harmonized system for describing the different mechanisms of relapse for fear and drug studies in this review. Accordingly, from this point forward, we will use *fear relapse* and *drug relapse* as umbrella terms for fear and drug return (for both human and animal models), with *renewal*, *spontaneous recovery*, *reinstatement*, *stress-induced relapse*, *reacquisition*, and *external disinhibition* being used according to their respective mechanisms (e.g., instead of “context-induced drug reinstatement” to describe relapse of drug-seeking following a change in context, we will use “drug renewal”). With these terms defined, we will now explore underlying and overlapping neural circuitry of relapse.

### Neural circuits of relapse

Several overlapping brain regions and circuits have been implicated in the learning and memory processes underlying fear and drug relapse, particularly of the amygdala, prefrontal cortex (PFC), hippocampus (HPC), and bed nucleus of the stria terminalis (BNST). Although the neural circuits guiding the conditioning and extinction of fear (LeDoux 2000; Maren 2001; Kim and Jung 2006; Sotres-Bayon et al. 2006; Herry et al. 2010; Janak and Tye 2015; Izquierdo et al. 2016) and drug-seeking (Robinson and Berridge 1993; Koob et al. 1998; Chao and Nestler 2004; Everitt and Robbins 2005; Koob and Volkow 2010; Janak and Tye 2015) have received considerable attention, research on the behavioral and brain mechanisms of extinction retention and relapse is increasing (Venniro et al. 2016; Mantsch et al. 2016; Khoo et al. 2017; Chen et al. 2017; Dong et al. 2017; Goode et al. 2018a; Farrell et al. 2018)—we will center our review on the roles of these structures and their circuits at the time of relapse expression. When applicable, we will also describe how these structures interact with other essential reward and aversion processing structures, such as the ventral tegmental area (VTA) and nucleus accumbens (NAc) (Shalev et al. 2002; Kalivas and McFarland 2003; Leri and Rizos 2005; Feltenstein and See 2008; Fuchs et al. 2008; Wise 2009; Stuber et al. 2010; Sun 2011)—connections between the VTA and NAc are also critically involved in drug relapse and are highlighted in other reports (Mahler and Aston-Jones 2012; Stefanik et al. 2013a; Gibson et al. 2018). We also appreciate that there are a number of important drug factors, sex, species, and developmental differences in the neural circuits under discussion in this review (Fuchs et al. 2005b; Kippin et al. 2005; Kim and Richardson 2010; Saunders and Robinson 2011; Bossert et al. 2013; Ganella and Kim 2014; Matsuda et al. 2015; Dejean et al. 2015; Den et al. 2015; Marchant et al. 2015; Swalve et al. 2016b; Becker and Koob 2016; Swalve et al. 2016a; Park et al. 2017; King et al. 2018; Zbukvic and Hyun Kim 2018), and these factors might influence the neurocircuitry of drug and fear relapse.

#### Amygdala

The expression of relapse is thought to rely, in part, on a loss of inhibition over the expression of excitatory conditioned responses (CRs) by amygdala neurons involved in representing CS–US and/or S–R–O associations (Quirk and Gehlert 2003; Pape and Pare 2010; Duvarci and Pare 2014; Tovote et al. 2015; Calhoon and Tye 2015; Namburi et al. 2016). In other words, relapse occurs because extinction memories no longer inhibit expression of Pavlovian or instrumental responses acquired during conditioning (Bouton 2014). Many decades of work have identified neural circuits in the amygdala involved in the conditioning and extinction of fear (Herry et al. 2008; Ciocchi et al. 2010; Senn et al. 2014). Accordingly, the expression of fear memories and ultimately relapse depends on outputs of (and connections within) the lateral nuclei (LA), basolateral nuclei (BLA), and central nuclei (CeA) of the amygdala (Paré et al. 2004; Izquierdo et al. 2016). The expression of extinction memories (especially extinguished fear memories) is thought to depend on the dominance of activity in “extinction” neurons of the BLA, along with local inhibition of BLA “fear” neurons and inhibition of CeA outflow via inhibitory intercalated (ITC) cells of the amygdala (Royer and Paré 2002; Likhtik et al. 2008; Ehrlich et al. 2009; Pinard et al. 2012; Asede et al. 2015; Tovote et al. 2015; Gafford and Ressler 2016) and/or the basomedial amygdala (BMA) (Adhikari et al. 2015). Thus, activity in the amygdala is associated with both the inhibition of extinguished fear and its relapse (and, in turn, may be localized to particular subregions and cell types) (Knapska and Maren 2009).

For example, increased neuronal activity in the amygdala is associated with both renewal and spontaneous recovery of extinguished fear (Hobin et al. 2003; Herry et al. 2008; Lin et al. 2011; Huang et al. 2013; Orsini et al. 2013; Tapias-Espinosa et al. 2018), as well as reinstatement of drug-seeking (Weiss et al. 2000; Ciccocioppo et al. 2001; Thiel et al. 2010; Polston et al. 2012; Hitora-Imamura et al. 2015). Similarly, gamma oscillations in the amygdala during extinction have been shown to correlate with levels of spontaneous recovery during retrieval (Courtin et al. 2014) and electrical stimulation of the amygdala can induce fear relapse (Kellett and Kokkinidis 2004). In turn, the amygdala (including its BLA/CeA regions) is essential for both fear and drug relapse, such that lesions of the amygdala prevent fear renewal (Herry et al. 2008), fear reinstatement (Laurent and Westbrook 2010), drug reinstatement (Meil and See 1997; Grimm and See 2000; Kantak et al. 2002; Fuchs and See 2002; Yun and Fields 2003; Wang et al. 2006; Rogers et al. 2008; Cummins et al. 2014; Li et al. 2015), and drug renewal (Fuchs et al. 2005a; Fuchs et al. 2007; Lasseter et al. 2011; Wells et al. 2013; Chaudhri et al. 2013; Stringfield et al. 2016; Pelloux et al. 2018). Additionally, inactivation studies suggest that the CeA plays a critical role in stress-induced drug relapse [(Shaham et al. 2000; McFarland et al. 2004) also, see (Leri et al. 2002; Wang et al. 2006; Yamada and Bruijnzeel 2011)]. When reinstatement is drug-primed, the role of amygdala is less clear (Fuchs and See 2002; Yun and Fields 2003; Fuchs et al. 2005a; Wang et al. 2006; Pockros-Burgess et al. 2014; Georgiou et al. 2015)—this may relate to the fundamental role of the amygdala in forming stimulus–reward or CS–behavior associations, while drug (US) exposure as a trigger may introduce other non-associative and amygdala-independent mechanisms. Nonetheless, once fear has relapsed, its re-extinction does not appear to require a fully functional BLA (Lingawi et al. 2017). These data suggest that the circuits required for extinction may change over time, though future forms of relapse may continue to rely on the amygdala.

Several neuromodulatory systems in the amygdala have also been implicated in regulating relapse. For example, dopamine (See et al. 2001; Tobin et al. 2013), serotonin (Pockros-Burgess et al. 2014), glucocorticoid (Stringfield et al. 2016), and opioid (Nygard et al. 2016) signaling in the BLA are all critical for drug relapse. Likewise, dopamine (Thiel et al. 2010) and glucocorticoid (Simms et al. 2012) signaling in the CeA is essential for stress-induced drug relapse, whereas corticotropin-releasing factor (CRF) signaling in CeA is required for drug reinstatement (Wang et al. 2006). Less is known about these systems in the amygdala during fear relapse, though many of these neurotransmitters have well-established roles in regulation of conditioned fear (de Quervain et al. 2009; Abraham et al. 2014; Bauer 2015; Andero 2015; Bocchio et al. 2016; Lee et al. 2016; Giustino and Maren 2018). That said, noradrenaline signaling in the BLA can enhance reinstatement of fear (Lin et al. 2011), while blockade of noradrenaline in CeA can attenuate stress-induced drug relapse (Leri et al. 2002). Cortisol treatment, which can potentiate reinstatement, enhances activity in the amygdala of male subjects (Kinner et al. 2018).

A number of amygdalar efferents have emerged as critical regulators of the expression of relapse (critical inputs to the amygdala are discussed in detail in relevant sections below). Immediate early gene expression in NAc-targeting BLA neurons is increased after cocaine reinstatement (McGlinchey et al. 2016). In turn, BLA projections to the NAc are necessary for drug reinstatement (Lee et al. 2013; Keistler et al. 2017). These findings are interesting in light of research implicating NAc-targeting cells of the BLA in the extinction of fear (Correia et al. 2016), as well as in the extinction of drug-seeking itself (Millan and McNally 2011; Keistler et al. 2017). Additionally, photostimulation of this pathway in conjunction with extinction reduces fear relapse [(Correia et al. 2016); also, see (Millan et al. 2017)]. Thus, BLA➔NAc cells appear important for the expression of extinction *and* relapse; however, it has not yet been fully reconciled how ablation of this extinction-promoting pathway facilitates relapse, unless there exist some functional heterogeneity and segregation in cells between BLA and NAc core/shell.

Amygdala projections to both cortical and subcortical targets are also important for fear and drug relapse (Vouimba and Maroun 2011). For example, BLA projections to the prelimbic (PL) region are necessary for cue-induced reinstatement of drug-seeking (Stefanik and Kalivas 2013). Relatedly, research indicates that PL-targeting cells of the BLA are engaged by and critical for driving fear expression to non-extinguished cues and contexts (Stevenson 2011; Sotres-Bayon et al. 2012; Senn et al. 2014; Burgos-Robles et al. 2017), suggesting roles for these circuits in fear and drug reinstatement in aversive contexts. Conversely, infralimbic (IL)-targeting BLA neurons also appear to be critical in regulating extinction of fear (Senn et al. 2014); inhibiting their activity may be associated with relapse, at least of conditioned fear. As will be discussed later, the BNST plays an important role in the regulation of stress-related relapse; CRF-releasing CeA➔BNST neurons are needed for stress-induced drug relapse (Erb et al. 2001). Beyond these circuits, BLA projections to the orbitofrontal cortex (OFC) (but not OFC to BLA) are required for drug reinstatement (Arguello et al. 2017). While not yet explored fully in the context of fear relapse, other critical fear-promoting amygdalar efferents, such as amygdala–brainstem (Penzo et al. 2014; Cheriyan et al. 2016) circuits, suggest similar roles for these circuits during relapse expression. Collectively, these data indicate that the amygdala is critical for encoding and expressing CS–US associations, whether those consist of CS–shock or CS–drug associations. Accordingly, the amygdala and its efferents are critically involved in relapse, as these circuits are an important site of conditioned and extinction memories.

#### Prefrontal cortex

The PL and IL regions of the prefrontal cortex (including homologous regions in humans) have been identified as critical regulators of fear and drug relapse (Giustino and Maren 2015; Moorman et al. 2015; Gourley and Taylor 2016). PL and IL contributions to learned behaviors are often found to be dissociable; more specifically, PL appears to facilitate conditioned behaviors and drive relapse (serving as a “go” structure), while IL functions as an inhibitory “stop” structure, promoting the expression of extinction memories and minimizing relapse. Nonetheless, there are several important caveats to these functions in both fear and drug behaviors, and we will address these complexities later on in the section.

In line with the “go” role of the PL, there is a large amount of evidence showing that PL is involved in promoting relapse in a broad sense. For example, fear renewal is associated with enhanced activity in PL neurons (Knapska and Maren 2009; Zelikowsky et al. 2013). Likewise, stress-induced drug relapse (via food deprivation) induces c-fos expression in PL (Shalev et al. 2003). Accordingly, PL inactivation has been shown to block relapse of both fear and drug-seeking, including fear renewal (Kim et al. 2013; Sharpe and Killcross 2015), drug reinstatement (drug-primed) (McFarland and Kalivas 2001; Capriles et al. 2003; Di Pietro et al. 2006; Stefanik et al. 2013b; Vassoler et al. 2013; Shen et al. 2014; Martín-García et al. 2014), drug reinstatement (cue-induced) (McLaughlin and See 2003; Di Pietro et al. 2006; Mashhoon et al. 2010) drug renewal (Fuchs et al. 2005a), and stress-induced drug relapse (Capriles et al. 2003; McFarland et al. 2004). Additionally, PL mediates fear expression in aversive contexts (Lemos et al. 2010; Kim et al. 2013; Rozeske et al. 2015; Cullen et al. 2015; Reis et al. 2016), suggesting a role for this structure in fear reinstatement. That said, activation of periaqueductal gray (PAG)-projecting neurons of PL (and of the anterior cingulate cortex) are associated with low fear and increased discrimination of shock-associated contexts (Rozeske et al. 2018), indicating some pathway-specific roles of PL efferents in regulating fear behaviors.

With regard to roles of particular neuromodulator systems in PL during relapse, dopamine signaling in PL is required for drug relapse, at least in some paradigms (Capriles et al. 2003; McFarland et al. 2004; See 2009; Liu et al. 2017; James et al. 2018; Wang et al. 2018b). When paired with a cocaine injection that is not sufficient to induce reinstatement by itself, increasing corticosterone signaling in PL is sufficient to induce reinstatement of drug-seeking (McReynolds et al. 2017). These effects are further dependent on endocannabinoids, such that corticosterone in PL reduces activity in local inhibitory interneurons in an endocannabinoid receptor-dependent process (McReynolds et al. 2017); the net result of which appears to facilitate activity of PL’s outputs. Stimulating serotonin 2C receptors in PL (and IL) blocks reinstatement of cocaine-seeking (Pentkowski et al. 2010). Additionally, infusions of oxytocin in PL appear to prevent stress-induced drug relapse (Han et al. 2014). Norepinephrine signaling in PL is also an important regulator of drug reinstatement (Schmidt et al. 2017; Otis et al. 2018). It is not yet known if these same systems affect fear relapse, but glucocorticoids (Reis et al. 2016), endocannabinoids, and serotonin (Fogaça et al. 2014; Almada et al. 2015) in PL are important for contextual fear expression, suggesting they may be involved in fear reinstatement.

Several studies now document the involvement of specific PL projections in the regulation of relapse. In aversive learning paradigms, amygdala-projecting neurons of PL are more strongly engaged by renewal as compared to extinction retrieval (Orsini et al. 2011; Knapska et al. 2012). The relapse of drug-seeking behavior also implicates the PL➔BLA circuit, which suggests that there is overlap in circuits regulating “go” behavior. For example, asymmetric inactivation of the PL and BLA has been shown to attenuate drug relapse (Mashhoon et al. 2010). Glutamate release from PL terminals to the NAc core is augmented after drug-primed reinstatement, suggesting that the NAc core mediates the influence of PL on relapse (McFarland et al. 2003; Kalivas 2009). Indeed, c-fos expression in the PL➔NAc core pathway is positively correlated with relapse of drug-seeking, and pharmacological disconnection of the PL and NAc core attenuates cue-induced reinstatement of cocaine-seeking behavior (McGlinchey et al. 2016). Moreover, it has been reported that optogenetic inhibition of PL terminals in the NAc core inhibits drug relapse and prevents drug cue-induced synaptic potentiation in the NAc (Stefanik et al. 2013b; Stefanik et al. 2016). The role of PL projections to NAc during fear relapse is not clear. In total, these data implicate the PL and its connections to the amygdala in relapse broadly, and PL-to-NAc cells in drug relapse in particular.

Consistent with the idea that IL serves as a “stop” structure, it has been shown that the IL is critically involved in the suppression of conditioned fear and reward-seeking behavior (Rhodes and Killcross 2004; Quirk and Mueller 2008; Peters et al. 2009; Sierra-Mercado et al. 2011); the essential role of IL in acquisition and consolidation of extinction memories has been an area of intense study (Quirk and Mueller 2008; LaLumiere et al. 2010; Gass and Chandler 2013; Van den Oever et al. 2013; Barker et al. 2014; Jasinska et al. 2015). Indeed, successful retrieval of extinguished fear is associated with enhanced activity in the IL [(Milad and Quirk 2002; Milad et al. 2007; Knapska and Maren 2009; Madsen et al. 2017); but, see (Chang et al. 2010; Fitzgerald et al. 2014)] whereas fear relapse is associated with a decrease in IL activation (Hefner et al. 2008; Knapska and Maren 2009; Hitora-Imamura et al. 2015; Kutlu et al. 2016). Interestingly, acute treatment with nicotine enhances the spontaneous recovery of extinguished contextual fear, which coincides with reductions in IL activation (Kutlu et al. 2016). Consistent with these reports, pharmacological *inactivation* of the IL results in a loss of extinguished fear and results in relapse (Laurent and Westbrook 2009; Hitora-Imamura et al. 2015; Marek et al. 2018a), while pharmacological *activation* of the IL impairs relapse and promotes extinction retrieval (Marek et al. 2018a)—provided that the spontaneous recovery of fear does not mask these effects (Do-Monte et al. 2015; Marek et al. 2018a). That said, reduced spontaneous recovery (in a two-way active avoidance paradigm) is associated with greater IL activity (Tapias-Espinosa et al. 2018). In drug-seeking paradigms, several studies demonstrate that lesion or inactivation of the IL results in the emergence (or even, enhancement) of drug reinstatement (for both cue- and drug prime-induced reinstatement), stress-induced drug relapse, and drug renewal across numerous drug types (Capriles et al. 2003; McLaughlin and See 2003; McFarland et al. 2004; Peters et al. 2008b). In turn, activation of IL diminishes drug reinstatement (Peters et al. 2008a; LaLumiere et al. 2012). Beyond lesions, blocking dopamine (D1) receptors in the IL appears to reduce drug relapse, at least for some forms of reinstatement (Cosme et al. 2018). Similarly, pre-shock blockade of these same receptors in IL blunted subsequent fear reinstatement (Hitora-Imamura et al. 2015). Thus, stress-induced drug relapse and fear reinstatement both may require dopaminergic input to the IL from the VTA (McFarland et al. 2004; Hitora-Imamura et al. 2015). Serotonin in the IL also appears to play a role in regulating drug relapse (but again, with similar results as in PL) (Pentkowski et al. 2010). There is still much to learn with regard to neuromodulatory systems in the PFC during relapse.

Differences in the function of IL and PL neurons are defined by the different efferent targets of these neurons (Pinard et al. 2012). For example, BLA-projecting neurons of the IL have been shown to exhibit increases in excitability following fear extinction—an effect that was not observed for BLA-targeting neurons of PL (Bloodgood et al. 2018). Successful retrieval of extinction is thought to rely on IL’s ability to engage extinction-promoting neurons in the amygdala, which may in turn inhibit fear- and perhaps drug-seeking-promoting cells (Paré et al. 2004; Herry et al. 2010). Other studies have shown amygdala-targeting cells of IL to be more strongly engaged when extinguished fear is successfully retrieved (Knapska et al. 2012). Nonetheless, the functional role of amygdala-targeting IL cells is not clear in context of drug relapse or successful retrieval of extinguished drug-seeking behaviors. PL projects predominantly to NAc core whereas IL projects almost exclusively to NAc shell (Berendse et al. 1992; Vertes 2004). Consistent with an inhibitory role of IL over relapse, it has been shown that stimulation of IL terminals in the shell of the NAc blocks morphine-induced reinstatement of CPP (Hearing et al. 2016). Interestingly, deep-brain stimulation of the shell of the NAc, which appears to induce increased c-fos expression in IL (but not PL), has been demonstrated to reduce reinstatement (drug-primed; cocaine) of drug-seeking (Vassoler et al. 2013). VTA inputs to the IL may provide the dopamine signaling that has been shown to be necessary for fear reinstatement, as this circuit is engaged during relapse (Hitora-Imamura et al. 2015). Also, expression of extinction of alcohol-seeking has been shown to induce activity in medial dorsal hypothalamus-targeting cells of IL (Marchant et al. 2010), suggesting a diversity of pathways may be engaged by extinction retrieval.

In contrast to a go/stop dichotomy, other works suggest that the PL and IL have similar roles in relapse. For example, relapse is associated with similar levels of immediate early gene expression in the IL and PL in some studies (Zavala et al. 2008; Koya et al. 2009; Bossert et al. 2011). While the aforementioned data may reflect competition between IL and PL during relapse, IL lesions have been found to impair (rather than enhance) relapse of drug-seeking (Koya et al. 2009; Rocha and Kalivas 2010; Bossert et al. 2011; Bossert et al. 2012; Vassoler et al. 2013; Pelloux et al. 2013). Similarly, targeted disruption of IL neurons that were active during exposure to the drug-taking context was shown to prevent later drug renewal [an effect that was not observed when disrupting IL neurons that were active in the extinction context (Bossert et al. 2011)]. Willcocks and McNally (2013) found that PL inactivation attenuated drug renewal. However, PL inactivation also potentiated reacquisition, an effect that may depend on the reintroduction of the reinforcer (but not the drug’s effects per se) (Willcocks and McNally 2013). This same study found no effect of IL inactivation on the expression of extinguished drug-seeking (though latencies were altered). Nonetheless, lesions of PL have in some cases been found to spare the relapse of drug-seeking (Bossert et al. 2011). Others have demonstrated limited effects of ablating PFC input to the BLA during reinstatement of alcohol-seeking (Keistler et al. 2017). Additionally, there is evidence that electrical stimulation of the IL induces long-lasting changes in the BNST (a region the IL heavily targets), and that these IL- and BNST-dependent changes can facilitate reinstatement (Reisiger et al. 2014).

One reason for these discrepancies may be the reciprocal connections between PL and IL (Vertes 2004; Hoover and Vertes 2007), such that one region may function abnormally in the absence of the other. Interestingly, projections from PL to IL appear to be greater than those from IL to PL (Marek et al. 2018b). Although it is not yet known if these circuits are involved at the time of relapse, recent work indicates that the extinction of conditioned fear engages projections of PL to IL and that photostimulation of these excitatory projections enhances the acquisition of extinction (Marek et al. 2018b). Photoinhibition of this pathway appeared to slow the rate of extinction acquisition (Marek et al. 2018b). These findings may also help explain other cases in which IL and PL neurons exhibit similar responses during the extinction of instrumental behaviors (Moorman and Aston-Jones 2015). Additionally, given that many of the non-overlapping results are found in drug-seeking paradigms, the complexity of behaviors associated with drug-seeking may contribute to cases in which there are a diversity of outcomes of prefrontal lesions on drug relapse (Gilmartin et al. 2014; Giustino and Maren 2015; Moorman et al. 2015; Gourley and Taylor 2016). Nevertheless, these data suggest a complicated, but important, role for the prefrontal cortex in both drug and fear relapse.

#### Hippocampus

Hippocampal activity in humans and other animals has been linked to forms of fear relapse that depend on contextual conditioning (reinstatement) or shifts in context (renewal) (Marinelli et al. 2007; Maren et al. 2013; Orsini et al. 2013; Lonsdorf et al. 2014; Jin and Maren 2015; Hermann et al. 2016; Wang et al. 2016; Scharfenort et al. 2016). Accordingly, HPC lesions or inactivation have been shown to disrupt both fear reinstatement (Frohardt et al. 2000) and fear renewal (Corcoran and Maren 2001; Corcoran and Maren 2004; Ji and Maren 2005; Corcoran et al. 2005; Hobin et al. 2006; Ji and Maren 2008; Marek et al. 2018a). Furthermore, inactivation of the hippocampus eliminates neuronal correlates of renewal in the amygdala (Maren and Hobin 2007). Additionally, the HPC is involved in novelty-detection processes that are often necessary to support relapse (Maren 2014). Interestingly, reacquisition of an inhibitory avoidance procedure was shown to rely on protein synthesis within the dorsal HPC (Cammarota et al. 2003); thus, contributions of the HPC to relapse may be quite broad. The direct role of the HPC in other forms of fear relapse (e.g., stress-induced) has not been explored; however, nicotine administration appears to enhance the spontaneous recovery of contextual fear, an effect which coincides with enhanced c-fos in the ventral HPC (Kutlu et al. 2016). Additionally, enhanced post-extinction amygdalar–hippocampal functional connectivity has been documented in individuals that exhibited robust spontaneous recovery of fear (as compared to weak spontaneous recovery) (Hermans et al. 2017). Moreover, pharmacogenetic activation of hippocampal cells that were active at the time of conditioning has been shown to result in fear relapse-like effects when tested for extinction memories (Yoshii et al. 2017).

Similar to its role in fear relapse, the HPC is engaged by and required for drug renewal and (cue- and drug-primed) reinstatement (Fuchs et al. 2005a; Cooper et al. 2006; Rogers and See 2007; Kufahl et al. 2009; Luo et al. 2011; Zhao et al. 2017; Ge et al. 2017), and in the relapse of conditioned place preference (Guan et al. 2014; Portugal et al. 2014; Assar et al. 2016). Expression of drug relapse is also affected by activity of the HPC during learning of the conditioned drug response (Martin-Fardon et al. 2008). Depending on the timing of its induction or ablation, adult hippocampal neurogenesis may protect against drug-primed reinstatement (Deschaux et al. 2014; Galinato et al. 2018), but its role in fear relapse has not been established [also, see (Seo et al. 2015)]. Similar to fear renewal, the ventral subiculum (vSUB) appears to be critically involved in context-induced drug reinstatement. It has been reported that inactivation of vSUB decreases renewal of heroin-seeking (Bossert and Stern 2014). Furthermore, electrical stimulation of vSUB has been shown to induce relapse itself (Taepavarapruk and Phillips 2003; Taepavarapruk et al. 2014). Both the dorsal and ventral HPC have been implicated in drug relapse, but this has not been entirely consistent across studies. For example, pharmacological inhibition of the ventral HPC, but not the dentate gyrus or posterior dorsal HPC, prevents drug renewal (Lasseter et al. 2010). This is in contrast to reports that have found effects of dorsal HPC manipulations on drug relapse: tetrodotoxin (but not anisomycin) in the dorsal HPC has been shown to prevent renewal in a drug-associated context (Ramirez et al. 2009).

Similar to the amygdala and PFC, dopamine plays an important role in HPC’s contributions to drug relapse (Khakpour-Taleghani et al. 2015; Haaker et al. 2015; Assar et al. 2016; Wang et al. 2018b). Additionally, infusions of oxytocin in dorsal HPC attenuate stress-induced drug relapse (Han et al. 2014)—a similar outcome as when oxytocin is infused into PL. Selective nicotinic receptor blockade in the ventral (but not dorsal) HPC disrupts the reinstatement of morphine–CPP (Wright et al. 2018). Contributions of these neurotransmitter systems to fear relapse have not yet been explored. That said, post-extinction (but pre-novelty exposure) adrenoceptor blockade in dorsal CA1 of the HPC blocks the extinction-enhancing effects of novel context exposure on measures of spontaneous recovery of fear and fear reinstatement (Chai et al. 2014; Liu et al. 2015).

Converging evidence suggests that the hippocampus strongly regulates relapse through its interactions with the prefrontal cortex. In particular, recent work has demonstrated that the HPC controls fear renewal by gating activity in the IL via feed-forward inhibitory mechanisms [(Marek et al. 2018a); also, see (Liu and Carter 2018)]. That is, glutamatergic HPC neurons target local inhibitory interneurons of the IL (as well as amygdala-targeting principal cells of IL); activation of HPC➔IL neurons engages strong inhibition of IL outputs, preventing their induction of extinction mechanisms. Pharmacogenetic activation or silencing of this pathway bidirectionally promoted or shunted relapse, respectively (Marek et al. 2018a). Other functional studies have shown co-activation of HPC➔IL and HPC➔PL cells during fear renewal (Wang et al. 2016), suggesting these circuits may work in tandem during relapse; however, the HPC appears to target PL to a lesser extent, so the HPC➔IL pathway may dominate the effects of HPC➔PFC stimulation. Due to this arrangement, it should be noted that functional dissociations in PL and IL may in part be explained by the extent of projections from ventral HPC to these structures (Jin and Maren 2015; Wang et al. 2016; Marek et al. 2018a). That said, reversible disconnection experiments have also suggested a role for connections between HPC and PL as being involved in fear relapse (Orsini et al. 2011; Fu et al. 2016).

Overlapping with fear relapse, addiction studies have found the HPC➔IL pathway to be critical to drug relapse insofar as renewal of heroin-seeking has been shown to engage HPC➔IL circuitry (Bossert et al. 2016; Wang et al. 2018a), and inactivation of HPC➔IL neurons prevents drug renewal (Wang et al. 2018a). The HPC➔PL pathway does not appear necessary for drug renewal (Bossert et al. 2016; Wang et al. 2018a). This may reflect a divergence of drug relapse from the fear relapse circuitry described above, or may highlight the larger role of HPC➔IL neurons in mediating context-dependent drug relapse, in particular. Moreover, Bossert et al. (2016) did not observe effects of anatomical disconnections of HPC–IL on drug renewal—however, given their targeting of vSUB, in particular, it is possible a majority of IL-targeting CA1 cells were capable of sustaining relapse. Additionally, it has been shown that protein kinase B (Akt) signaling along the HPC➔PFC pathway critically regulates the reinstatement of morphine–CPP (Wang et al. 2018b). Overall, these data suggest a complicated relationship between HPC, IL, and PL, but consistently suggest a regulatory role of HPC on PFC-dependent relapse.

Perhaps in conjunction with its interactions in the PFC, the HPC also is thought to regulate relapse via its strong connections to the amygdala (Maren and Hobin 2007; Orsini et al. 2011). In particular, photoinhibition of hippocampal terminals in CeA attenuates renewal (Xu et al. 2016). Interestingly, photoinhibition of HPC➔CeA cells did not affect contextual fear (Xu et al. 2016), suggesting that this pathway may not contribute to reinstatement (though this remains untested). Conversely, photoinhibition of HPC➔BLA reduced contextual fear, but not renewal (Xu et al. 2016), also suggesting that HPC➔BLA neurons may facilitate reinstatement (though untested). These results are interesting in light of disconnection studies targeting ventral HPC and BLA (Orsini et al. 2011); these data suggest roles for indirect connections and/or reciprocal feedback from BLA to HPC in relapse. Relatedly, and while connections are far more extensive between BLA and ventral HPC, disconnection of BLA and dorsal HPC has been shown to attenuate renewal of drug-seeking (though it remains unclear the directionality of this relationship) (Fuchs et al. 2007). Drug relapse may also broaden relapse-regulating circuits of HPC. Indeed, NAc shell-targeting vSUB cells have been shown to be engaged during renewal of heroin-seeking, and disconnection of these regions attenuates the renewal of heroin-seeking (Bossert et al. 2016). Dual-virus pharmacogenetic techniques have further shown that inactivation of vSUB projections to NAc shell prevents renewal of alcohol-seeking after punishment-imposed abstinence (Marchant et al. 2016). While these projections to the NAc may indirectly regulate VTA-dependent relapse, others have also shown that drug renewal incorporates dorsal HPC projections to the lateral septum (McGlinchey and Aston-Jones 2018), including a serial relay of HPC➔lateral septum (LS)➔VTA cells (Luo et al. 2011). In sum, the role of the HPC in relapse is heavily tied to its connections with amygdala, PFC, and NAc.

#### Bed nucleus of the stria terminalis

Considerable work indicates that the BNST is involved in fear and drug relapse that depends on contextual fear and stress. This may be due to the broad role of the BNST in processing uncertain threats (Davis et al. 2010; Avery et al. 2016; Gungor and Paré 2016), particularly in situations in which there is uncertainty about *when* an aversive event will occur (Goode and Maren 2017; Goode et al. 2018b). Indeed, unsignaled and unpredictable footshock exposure (often used for stress-induced drug relapse or fear reinstatement) induces c-fos expression in the BNST (Erb et al. 2004; Lin et al. 2018). Furthermore, contextual fear expression in the aftermath of footshock is associated with activity in the BNST (Lemos et al. 2010; Luyten et al. 2012; Ali et al. 2012). At the time of relapse, fear reinstatement has been linked with increased activation in the human BNST (Scharfenort and Lonsdorf 2016). In animals, stress-induced drug relapse is associated with increased c-fos expression in the BNST (Zhao et al. 2006; Schank et al. 2015). Additionally, acute food deprivation, which has been shown to induce stress-induced drug relapse, triggers an increase in c-fos levels in subregions of the BNST (Shalev et al. 2003). Stress-induced drug relapse produced by systemic administration of norepinephrine (NE) or the pharmacological stressor, yohimbine, is also associated with increased activity in the BNST (Brown et al. 2011; Funk et al. 2016). Moreover, drug reinstatement has also been shown to increase c-fos in the BNST (Jupp et al. 2011).

In turn, permanent and reversible lesions of the BNST disrupt contextual fear expression (including behavioral and autonomic readouts) and subsequent fear reinstatement (Sullivan et al. 2004; Waddell et al. 2006; Waddell et al. 2008; Goode et al. 2015b). Recent work suggests that contextual conditioning and its expression can be made independent of an intact BNST, if training occurs such that shock onset occurs early on and in a predictable manner (Hammack et al. 2015). Thus, there may be cases in which reinstatement is independent of the BNST, although this remains to be tested. In cases where the test context is not excitatory, but contextual information is nonetheless important (such as with fear renewal), lesions of the BNST spare relapse (Goode et al. 2015b); this again suggests a reliance on the BNST in stress-dependent forms of relapse. Contributions of the BNST to the expression of other forms of relapse have not yet been extensively explored. That said, concurrent neuropeptide Y receptor (particularly, Y2 receptor) antagonism in the BNST has been shown to impair extinction acquisition as compared to animals treated with Y2 receptor agonists or saline in the BNST; these effects mirror levels of spontaneous recovery of this extinguished fear at remote time points (Verma et al. 2018). In the absence of extinction, Y2 receptor agonism appears to weaken incubation of the fear CS memory (Verma et al. 2018).

As with fear relapse, permanent and reversible lesions of the BNST block various forms of stress-induced drug relapse, including through the use of footshock (McFarland et al. 2004), swim stress (Briand et al. 2010), and pharmacological stressors (Buffalari and See 2011). Interestingly, reversible inactivation of the BNST also blocks drug reinstatement (Buffalari and See 2011)—fear reinstatement by non-extinguished fear cues has not yet been demonstrated to rely on the BNST. The absence of an effect of BNST inactivation on fear renewal (Goode et al. 2015b) may suggest a limited role for the BNST in drug renewal; nevertheless, this possibility (along with the BNST’s role in other forms of drug relapse) is still unaddressed.

Stress-related neurotransmitter systems, such as CRF, NE, and pituitary adenylate cyclase-activating polypeptide (PACAP), are known to make significant contributions to relapse by acting within the BNST (Harris and Winder 2018). For example, triggers for stress-induced fear and drug relapse, as well as drug reinstatement itself, have been shown to coincide with increases in CRF mRNA in dorsal (but not ventral) regions of the BNST (Shalev et al. 2001; Funk et al. 2006). In turn, CRF antagonism in the BNST (but not amygdala) prevents stress-induced drug relapse (Erb and Stewart 1999; McReynolds et al. 2014). Induction of NE signaling in BNST can induce drug relapse (Vranjkovic et al. 2014), while adrenoceptor antagonism in the BNST prevents stress-induced drug relapse in a receptor- and dose-dependent manner (Leri et al. 2002). Blockade of either NE or CRF signaling in BNST prevents stress-induced drug relapse of CPP (Wang et al. 2001; Wang et al. 2006). Similar effects were shown for drug relapse when hypocretin/orexin antagonists or cannabinoid antagonists were infused into the BNST (Reisiger et al. 2014; Ubaldi et al. 2016). Although not yet demonstrated for fear relapse, activation of CRF or PACAP signaling within the BNST has been shown to induce relapse of drug-seeking (Erb and Stewart 1999; Miles et al. 2018b; Miles et al. 2018a).

With regard to the contributions of BNST efferents in relapse, forced-swim stress, which has been shown to induce drug reinstatement, is associated with increased c-fos expression in VTA-targeting cells of the BNST (Briand et al. 2010). Additionally, pharmacological inactivation of the BNST is associated with decreased c-fos expression in the VTA, CeA, and NAc core (Briand et al. 2010). Furthermore, pharmacological disconnection studies indicate that NE-activated CRF-releasing VTA projection neurons of the BNST are required for stress-induced drug relapse (Vranjkovic et al. 2014). BNST sends and receives extensive projections to and from the amygdala, PFC, HPC, VTA, and NAc, suggesting there is still much to uncover with regard to its potential contributions to fear and drug relapse. Overall, however, the BNST is clearly a site of stress-related relapse regulation.

## Conclusions: Converging neural circuits for fear and drug relapse

**Fig. 2:**
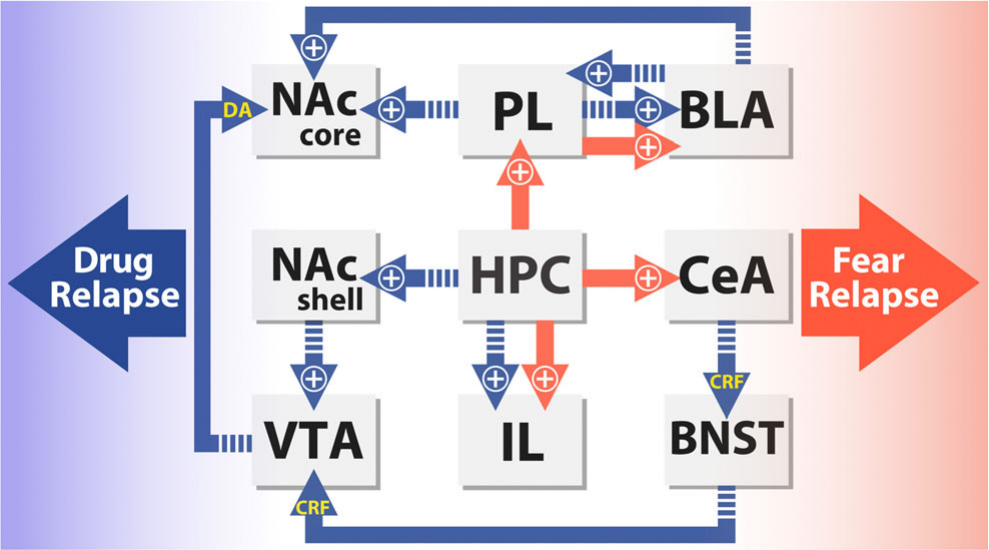
Arrows indicate common and divergent regions and neural pathways that have been demonstrated as directly engaged by and/or are required for expression of either drug relapse (blue dashed arrows) or for fear relapse (red arrows) after extinction. Relapse circuits are drawn from studies that are not limited to any particular drug type (cocaine, methamphetamine, etc.) or relapse scenario (spontaneous recovery, renewal, etc.). “+” symbols indicate excitatory (glutamatergic) pathways. Arrows labeled with “DA” and “CRF” denote dopaminergic or corticotrophin-releasing factor-releasing relapse circuits, respectively. Region abbreviations: nucleus accumbens core (NAc core); nucleus accumbens shell (NAc shell); ventral tegmental area (VTA); prelimbic cortex (PL); hippocampus (HPC); infralimbic cortex (IL); basolateral amygdala (BLA); central amygdala (CeA); bed nucleus of the stria terminalis (BNST)

Based on the foregoing review, it is apparent that common neural circuits regulate the maintenance and relapse of extinguished responding to fear and drug CSs (Fig. 2; also, see Table 1). Importantly, connections between the PFC and the amygdala are a key point of convergence in the neural circuitry for fear and drug relapse. In particular, excitatory outputs of the PL division of the PFC to the amygdala are important for the expression of both fear and drug relapse. Context-mediated forms of fear and drug relapse (e.g., renewal and reinstatement) invoke the hippocampus and its circuitry, with relapse engaging excitatory IL-projecting HPC neurons in both fear and drug relapse. Furthermore, stress (e.g., reinstatement and stress-induced relapse) recruits the BNST and its circuits during both fear and drug relapse. Additionally, dopaminergic signaling in the PFC and norepinephrine in the amygdala appear to critically regulate both fear and drug-seeking behaviors during relapse. Although the neural circuits mediating fear relapse are centered on the PFC, HPC, and amygdala, drug relapse broadens the roles of these circuits to include the VTA and NAc. Differences in the circuits of fear and drug relapse may relate to the nature of the behaviors being examined (e.g., freezing vs. active lever pressing) as well as the impact of drug use (or abstinence) on the circuitry. Further insight into the common neurocircuitry of relapse may be gleaned by examining relapse of aversively motivated instrumental behaviors, such as after the extinction of active avoidance. Additionally, further work should consider whether manipulations that attenuate drug relapse can reduce fear relapse (and vice versa) in the same animals. To conclude, overlapping neural circuits of fear and drug relapse may explain, in part, the high comorbidity of fear-related pathologies and addiction. The characterization of the brain circuits underlying relapse in preclinical models may help inform the development of therapeutic interventions for psychiatric disorders in humans.

**Table 1:** Additional information based on studies examining relapse circuits of the amygdala, prefrontal cortex, hippocampus, and BNST (summarized in Fig. 2). Dashed marks indicate cases in which results of the manipulation are currently unknown

Circuit	Manipulations		Result for drug relapse	Result for fear relapse
BLA➔PL	Inhibition	Optogenetics	Decreased ↓	–
BLA➔NAc (core)	Inhibition	Pathway-specific cell ablation	Decreased ↓	–
CeA➔BNST	Inhibition of CRF	Pharmacological disconnection	Decreased ↓	–
PL➔NAc (core)	Inhibition	Optogenetics	Decreased ↓	–
PL➔BLA	Inhibition	Pharmacological disconnection	Decreased ↓	Decreased ↓
HPC➔IL	Inhibition	Optogenetics/DREADDs	Decreased ↓	Decreased ↓
	Excitation	DREADDs	–	Increased ↑
HPC➔PL	Inhibition	Pharmacological disconnection	–	Decreased ↓
		Optogenetics	Null result	–
HPC➔CeA	Inhibition	Optogenetics	–	Decreased ↓
HPC➔NAc (shell)	Inhibition	Pharmacological disconnection/KORDs	Decreased ↓	–
BNST➔VTA	Inhibition of CRF	Pharmacological disconnection	Decreased ↓	–
